# Impact of concurrent right ventricular myocardial infarction on outcomes among patients with left ventricular myocardial infarction

**DOI:** 10.1038/s41598-020-58713-0

**Published:** 2020-02-03

**Authors:** Huocheng Liao, Qiuyue Chen, Lin Liu, Sigan Zhong, Huazhao Deng, Chun Xiao

**Affiliations:** The Third People Hospital of Huizhou, The Affiliated Hospital of Guangzhou Medical University, Huizhou, Guangdong China

**Keywords:** Interventional cardiology, Disease-free survival

## Abstract

To compare in-hospital outcomes between left ventricular myocardial infarction (LVMI) patients with and without right ventricular myocardial infarction (RVMI). Patients with acute ST-segment elevation MI (STEMI) undergoing primary percutaneous coronary intervention (PCI) were enrolled and divided into LVMI with and without RVMI groups. Between-group differences and in-hospital outcomes were compared. Compared to patients without RVMI, patients with RVMI were more likely to be male, have higher body mass index, serum levels of C-reactive protein (8.9 ± 2.4 vs 6.2 ± 2.1 mg/dL), B-type natriuretic peptide (1295 ± 340 vs 872 ± 166 pg/mL) and cardiac troponin-I (8.6 ± 2.9 vs 5.2 ± 2.1 ng/mL), and have diabetes (36.3% vs 3.4%) and dyslipidemia (53.4% vs 48.1%). Patients with RVMI had lower left and right ventricular ejection fraction (50.5 ± 5.6% vs 53.4 ± 3.8% and 33.6 ± 2.9% vs 45.7 ± 2.0%), but had higher mean pulmonary artery pressure (30.6 ± 3.3 vs 23.8 ± 3.1 mm Hg). Compared to patients without RVMI, patients with RVMI had higher odds of in-hospital all-cause mortality (4.1% vs 1.0%) and new onset acute heart failure (3.4% vs 1.0%). After adjusted for confounding factors, LVMI with RVMI remained independently associated with composite outcomes, with odds ratio 1.66 (95% confidence interval 1.39–2.04). Compared to isolated LVMI patients, those with concomitant RVMI have higher odds of in-hospital complications, particularly all-cause mortality and new onset acute heart failure.

## Introduction

Acute myocardial infarction (AMI) remains a leading cause of morbidity, mortality and medical lost around the world including China^1–4^. Heart failure (HF) is a common and severe complication of AMI^5,6^. Different mechanisms contribute to HF development including impaired ventricle contraction, myocardium stunning or/and hibernation, mechanical implications and among others^7–9^. Of note, the survival rate of patients with symptomatic HF is less than 50%^9^. In addition, patients with HF are also more likely to re-hospitalize than those without HF^10–12^. Therefore, it is clinically important and relevant to prevent HF development and progress among MI patients. Furthermore, elucidating the mechanisms contributing to HF development post-MI is also essential for developing targeted intervention in the future.

Right ventricular MI (RVMI) commonly accompanies with left ventricular MI (LVMI), and the impairment of RV function is predominantly determined by the size and location of the infarction^13–15^. RV function and contraction can be altered in the setting of ischemic injury. In addition, the change of pulmonary circulation resistance due to LVMI can also influence RV function, which in turn leads to decreased RV contraction^15–17^. Currently, limited evidence indicates that compared to LVMI patients without RVMI, their counterparts with RVMI has poorer outcomes. However, the underlying mechanisms are multifactorial and not fully clear yet.

In our current study, we conducted a retrospective study to compare in-hospital outcomes between LVMI patients with and without RVMI. In addition, we also tentatively explored the underlying mechanisms associated with the poorer outcomes in LVMI patients with RVMI. We considered that data from our current study would provide novel insights into the impact of RVMI on outcomes.

## Methods

### Study participants enrollment

Our current study was approved by the Clinical Research Ethics Committee of the Third People’s Hospital of Huizhou. Since this is a retrospective study, the need for written informed consent was waived by the Clinical Research Ethics Committee. All research were performed in accordance to the standard guideline of Health Insurance Portability and Accountability Act of China. Patients hospitalized in our department between January of 2017 to December of 2018 were screened and patients with acute ST-segment elevation MI (STEMI) undergoing primary percutaneous coronary intervention (PCI) were enrolled and divided into LVMI with and without RVMI groups.

### Baseline characteristics collection

Demographics including age, gender and body mass index (BMI) calculated by weight in kilogram divided by height in squared meters; AMI risk factors including smoking status, hypertension, dyslipidemia and diabetes mellitus; laboratory parameters including fasting blood glucose, total cholesterol, C-reactive protein (CRP), B-type natriuretic peptide (BNP), cardiac troponin-I (CTn-I) and creatinine were collected from electronic medical record. Baseline serum creatinine was used to calculate estimated glomerular filtration rate (eGFR) using the MDRD formula. In addition, data of echocardiography 24 hour after PCI were also obtained. In specific, the electrocardiographic diagnostic criteria of RVMI were as follows: V4R showed loss of R-wave height, significant ST elevation (>0.5 mm; ST segment > R wave) and hyper-acute T wave (very large T wave given amplitude of QRS complex).

### Characteristics of PCI

Characteristics of PCI including femoral artery access, antiplatelet medication loading, lesion locations, number and type of stents implanted, volume of contrast used, initial and final TIMI flow score, collateral circulation, number of disease vessels, and the duration symptom onset to PCI were extracted from electronic medical record. All these data collection were done by two independent investigators.

### Study outcomes

The study outcomes assessed in our current study were composite of in-hospital all-cause mortality, new onset acute HF, and recurrent MI. All these events were adjudicated by independent cardiologists.

### Statistical analysis

Continuous variables were presented as mean ± SD and categorical variables were presented by proportion and number. Using the chi-square test for comparison of categorical variables and t-student for continuous variables. Logistic regression analysis was performed to evaluate the association of LVMI plus RVMI and composite outcomes, and patients without RVMI was served as reference group. Statistical software SPSS24.0 was used to perform statistical analysis. P value < 0.05 was considered as statistical significance.

## Results

### Baseline characteristics comparisons

**Figure 1:**
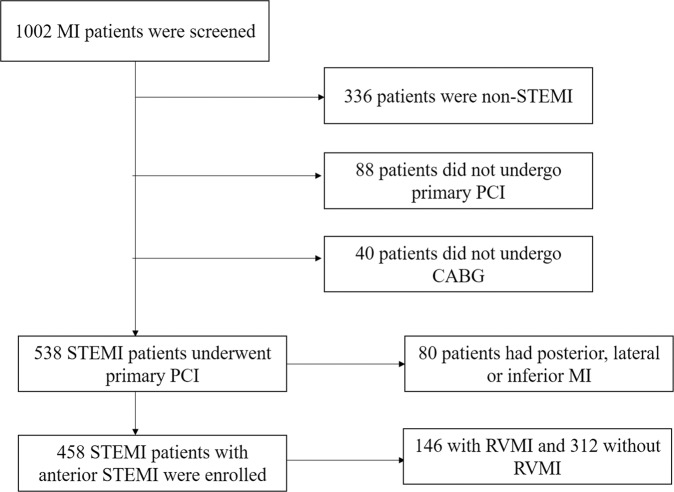
Study schematic.

A total of 458 patients were enrolled (Fig. 1) and patients were divided into two groups based on with (n = 146) and without (n = 312) RVMI. As presented in Table 1, compared to patients without RVMI, patients with RVMI were more likely to be male (65.1% vs 57.7%), have higher BMI (25.8 ± 7.2 vs 23.1 ± 6.5 kg/m^2^), higher serum levels of FBG (99.6 ± 13.4 vs 94.0 ± 12.5 mg/Dl), CRP (8.9 ± 2.4 vs 6.2 ± 2.1 mg/dL), BNP (1295 ± 340 vs 872 ± 166 pg/mL) and CTn-I (8.6 ± 2.9 vs 5.2 ± 2.1 ng/mL), and have diabetes mellitus (36.3% vs 3.4%) and dyslipidemia (53.4% vs 48.1%). Among the 146 patients with RVMI, 128 (87.7%) patients showed loss of R-wave height, significant ST elevation (>0.5 mm; ST segment > R wave) and hyper-acute T wave in their first electrocardiography, and 18 (12.3%) patients showed loss of R-wave height, significant ST elevation (>0.5 mm; ST segment > R wave) in their first electrocardiography.

**Table 1:** Baseline characteristics comparisons.

Variables	LVMI with RVMI (n = 146)	LVMI without RVMI (n = 312)
Age (years)	51.4 ± 16.5	52.8 ± 17.2
Male, n (%)	95 (65.1)*	180 (57.7)
Current smoker, n (%)	44 (30.1)	95 (30.4)
Body mass index (kg/m^2^)	25.8 ± 7.2*	23.1 ± 6.5
FBG (mg/dL)	99.6 ± 13.4*	94.0 ± 12.5
Total cholesterol (mmol/L)	5.1 ± 0.9	5.0 ± 1.1
CRP (mg/dL)	8.9 ± 2.4*	6.2 ± 2.1
BNP (pg/mL)	1295 ± 340*	872 ± 166
CTn-I (ng/mL)	8.6 ± 2.9*	5.2 ± 2.1
Creatinine (umol/L)	66.4 ± 18.7	65.2 ± 19.6
eGFR (ml/min/1.73 m^2^)	90.7 ± 15.4	88.6 ± 17.2
Hypertension, n (%)	82 (56.2)	175 (56.1)
Diabetes mellitus, n (%)	53 (36.3)*	101 (31.4)
Dyslipidemia, n (%)	78 (53.4)*	150 (48.1)

FBG, fasting blood glucose; eGFR, estimated glomerular filtration rate; CRP, C-reactive protein;

*P < 0.05 versus LVMI without RVMI group.

### Comparisons of PCI characteristics

As presented in Table 2, compared to patients without RVMI, patients with RVMI had more likely to have right coronary artery lesions (100% vs 28.2%), and had higher number of stents implanted (2.6 ± 0.6 vs 2.1 ± 0.7) and higher volume of contrast used (169 ± 55 vs 132 ± 40 ml). No significant differences in femoral artery access, antiplatelet medications loading, other lesion locations, type of stents implanted, initial and finial TIMI flow score, collateral circulation, number of disease vessels and time to PCI were observed.

**Table 2:** Comparisons of PCI characteristics.

Variables	LVMI with RVMI (n = 146)	LVMI without RVMI (n = 312)
Femoral artery access, n (%)	65 (44.5)	143 (45.8)
Antiplatelet loading, n (%)	124 (84.9)	266 (85.3)
RCA Lesion locations		
Left main, n (%)	19 (13.0)	46 (14.7)
LAD, n (%)	126 (86.3)	270 (86.5)
LCX, n (%)	50 (34.2)	105 (33.7)
RCA, n (%)	146 (100)*	88 (28.2)
Proximal, n (%)	72 (49.3)*	31 (9.9)
Middle, n (%)	46 (31.5)*	28 (9.0)
Distal, n (%)	28 (19.2)*	29 (9.3)
Initial TIMI flow grade		
Grade 3 Grade 2 Grade 1 Grade 0	0 0 8 (5.5) 138 (94.5)	0 0 17 (5.4) 295 (94.6)
Final TIMI flow grade		
Grade 3 Grade 2 Grade 1 Grade 0	143 (97.9) 3 (2.1) 0 0	310 (99.4) 2 (0.6) 0 0
Collateral circulation, n (%)	16 (10.9)	37 (11.8)
Number of disease vessels	2.1 ± 0.8	2.0 ± 0.6
Drug-eluting stent, n (%)	142 (97.3)	304 (97.4)
Volume of contrast (ml)	169 ± 55*	132 ± 40
Time to PCI (minutes)	77.6 ± 32.1	75.8 ± 30.9

LAD, left anterior descending; LCX, left circumflex; RCA, right coronary artery; *P < 0.05 versus without RVMI group.

### Comparisons of echocardiography after PCI

**Figure 2:**
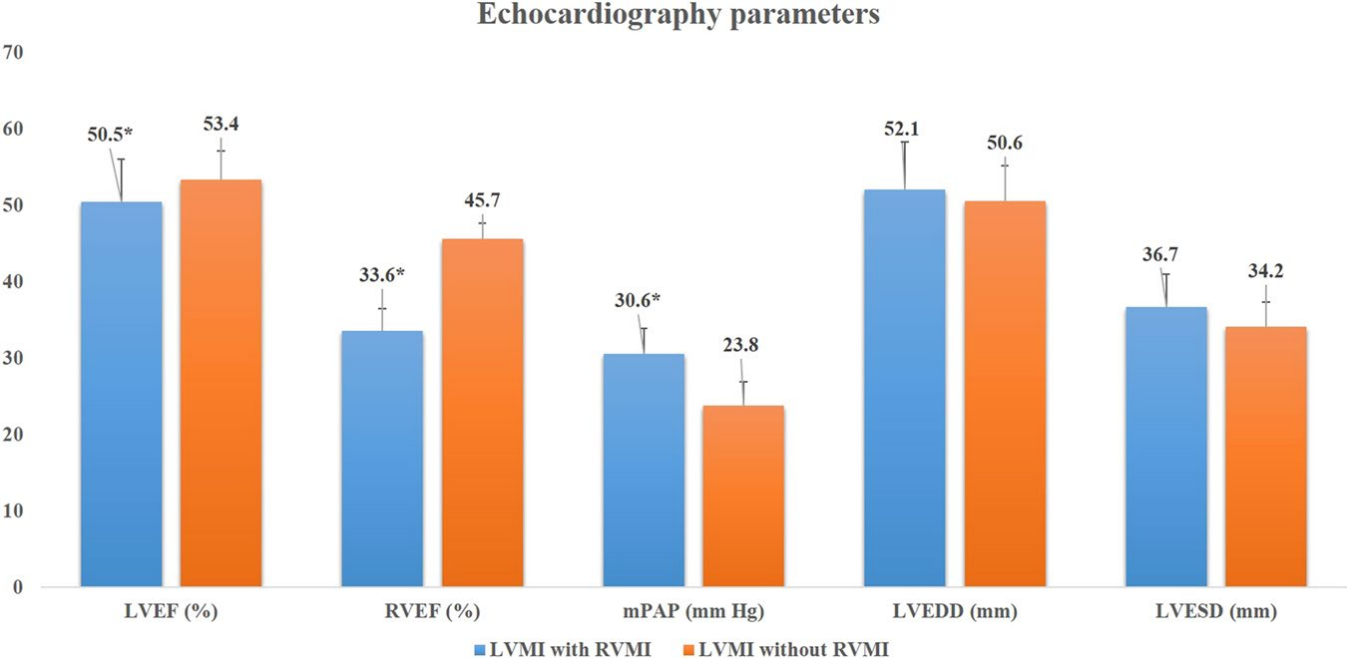
Comparisons of echocardiography parameters between LVMI patients with and without RVMI.

Echocardiography parameters 24 hour after PCI were performed between LVMI with and without RVMI. As shown in Fig. 2, compared to those without RVMI, patients with RVMI had lower left and right ventricular ejection fraction (LVEF [50.5 ± 5.6% vs 53.4 ± 3.8%] and RVEF [33.6 ± 2.9% vs 45.7 ± 2.0%]), but had higher mean pulmonary artery pressure (mPAP 30.6 ± 3.3 vs 23.8 ± 3.1 mm Hg). No significant differences in other parameters were observed.

### Comparisons of in-hospital outcomes

As presented in Table 3, compared to patients without RVMI, patients with RVMI had higher odds of in-hospital all-cause mortality (4.1% vs 1.0%) which were mainly driven by cardiovascular death (3.4% vs 0.3%), new onset acute HF (3.4% vs 1.0%) and composite outcomes (10.3% vs 3.8). No significant differences in other outcomes were observed.

**Table 3:** Comparisons of in-hospital outcomes.

Outcomes	LVMI with RVMI (n = 146)	LVMI without RVMI (n = 312)
All-cause mortality, n (%)	6 (4.1)	3 (1)*
Cardiovascular death, n (%)	5 (3.4)	1 (0.3)*
Respiratory failure, n (%)	1 (0.7)	2 (0.7)
New onset acute HF, n (%)	5 (3.4)	3 (1.0)*
Cardiogenic shock, n (%)	2 (1.4)	2 (0.6)
Recurrent MI, n (%)	2 (1.4)	4 (1.3)
Composite, n (%)	15 (10.3)	12 (3.8)*

HF, heart failure; MI, myocardial infarction; *P < 0.05 versus without RVMI group.

### Associations of LVMI with RVMI and composite outcomes

In order to evaluate the association of LVMI with RVMI and composite outcomes, patients with LVMI without RVMI were served as reference group. As presented in Table 4, in unadjusted model, the odds ratio (OR) of LVMI with RVMI for composite outcome was 2.21 with 95% confidence interval (CI) was 1.86–2.93. After stepwise adjusted for potential confounding factors, LVMI with RVMI remained independently associated with composite outcomes, with OR 1.66 (95% CI 1.39–2.04).

**Table 4:** Associations of LVEMI with RVMI and composite outcomes.

Independent variables	Odds ratio	95% Confidence interval
Unadjusted (LVMI with RVMI vs LVMI without RVMI)	2.21	1.86–2.93
Model 1	2.04	1.62–2.67
Model 2	1.85	1.50–2.35
Model 3	1.66	1.39–2.04

Model 1, adjusted for age, male gender, body mass index; model 2, variables in Model plus smoking status, diabetes mellitus, hypertension, dyslipidemia, C-reactive protein, estimated glomerular filtration rate, B-type natriuretic peptide and cardiac troponin-I; model 3, further adjusted for number of stents implanted, left ventricular ejection fraction and mean pulmonary artery pressure.

## Discussion

To our knowledge, this is the first few studies to evaluate the influence of RVMI on outcomes among STEMI patients undergoing PCI in China. Data from our current study suggests that compared to LVMI patients without RVMI, LVMI patients with RVMI have poorer in-hospital outcomes even after adjusted for potential confounding factors, indicating that future studies should be conducted to evaluate the underlying mechanisms attributed to RVMI-related outcomes in patients with LVMI.

Despite advancement in primary prevention and peri-PCI management have been achieved in the last decades, the morbidity and mortality rates post-AMI remains high, particularly in China due to the lacking health resource^1,2^. Notably, patients with LVMI are more likely to experience adverse cardiovascular events compared to patients with other area of MI (e.g. inferior MI or RVMI)^17^. The underlying mechanisms are predominantly attributed to a larger area of cardiomyocytes necrosis and apoptosis after left anterior descending artery occlusion. Compared to isolated LVMI, the incidence of isolated RVMI is relatively lower and the reasons for this difference are unclear^18,19^. Nevertheless, limited evidence has suggested that patients with isolated RVMI also had higher rates of adverse cardiovascular events. For example, Muhammad *et al*. reported that in acute RVMI patients, in-hospital complications occurred in 77% of patients, and cardiogenic shock was the commonest complication followed by acute left ventricular failure (LVF)^20^. In addition, 38 patients died. They concluded that frequency of complications is higher and cardiogenic shock is the most common complication in acute RVMI patients. Extending findings from prior report, Alhamshari *et al*.^13^ enrolled 105 patients and their findings also confirmed that RVMI not only increased in-hospital complications but also increased 2-years’ risk of HF development. Consistent with prior studies, our current analyses also found that patients with RVMI had higher in-hospital complications despite these patients undergoing primary PCI. The advantages of our current study are our relatively larger sample size and all patients enrolled in our study underwent optimal invasive therapy in terms of primary PCI. Evidence from these studies together support the notion that RVMI is a significant predictor for in-hospital complications.

The mechanisms attributed to the influence of RVMI on outcomes have been investigated before although not yet fully clearly. To our knowledge, two potential reasons may explain our current findings. First, RVMI may compromise left ventricular cardiac output due to decreased RV contraction and increased mPAP^21,22^. Indeed, in our current study, we also observed that compared to those without RVMI, patients with RVMI had lower right ventricular ejection fraction and higher mPAP, and all these hemodynamic changes in turn could impair left ventricular function and cardiac output. Second, RV function impairment could result in peripheral venous congestion (e.g. intestinal tract edema). Notably, intestinal tract edema is associated with increased systemic inflammation due to bacterial migration from damaged intestinal wall and increased toxin absorption^23–25^. Indeed, in our current study, we also observed that patients with RVMI had significantly higher C-reactive protein level. Once again supports the notion that enhanced inflammatory reaction may contribute to adverse outcomes in patients with RVMI.

There are some limitations of our current study needed to address. First of all, this is a retrospective study and many inherent biases could influence the association of RVMI and outcomes. However, findings from our study provided insight into the association of RVMI and in-hospital outcomes among STEMI patients undergoing primary PCI. Second, despite we have adjusted for different confounding factors, undetected and unmeasured confounding factors could still exist. Third, current study is from China’s population and whether findings from our study could extrapolate to other population groups are unknown. Last but not the least, we only evaluated the association of RVMI and in-hospital outcomes. Future studies are needed to evaluate the association of RVMI and long term outcomes.

## Conclusion

In conclusion, our study suggests that among STEMI patients undergoing primary PCI, compared to isolated LVMI patients, those with concomitant RVMI have higher odds of in-hospital complications, particularly all-cause mortality and new onset acute HF. Future studies are necessary to investigate how to better improve the outcomes of these patients.
